# 2′-Fucosyllactose Supplementation Improves Gut-Brain Signaling and Diet-Induced Obese Phenotype and Changes the Gut Microbiota in High Fat-Fed Mice

**DOI:** 10.3390/nu12041003

**Published:** 2020-04-05

**Authors:** Sunhye Lee, Michael Goodson, Wendie Vang, Karen Kalanetra, Daniela Barile, Helen Raybould

**Affiliations:** 1 Department of Anatomy, Physiology, and Cell Biology, University of California Davis School of Veterinary Medicine, Davis, CA 95616, USA; suhlee@ucdavis.edu (S.L.); mlgoodson@ucdavis.edu (M.G.); whvang@ucdavis.edu (W.V.); 2 Department of Food Science and Technology, University of California Davis, Davis, CA 95616, USA; kmkalanetra@ucdavis.edu (K.K.); dbarile@ucdavis.edu (D.B.)

**Keywords:** 2′-fucosyllactose, gut microbiota and metabolites, gut-brain axis, intestinal epithelial permeability, inflammation, diet-induced obesity

## Abstract

Obesity is characterized by fat accumulation, chronic inflammation and impaired satiety signaling, which may be due in part to gut microbial dysbiosis. Manipulations of the gut microbiota and its metabolites are attractive targets for obesity treatment. The predominant oligosaccharide found in human milk, acts as a prebiotic with beneficial effects on the host. However, little is known about the beneficial effects of 2′-FL in obesity. The aim of this study was to determine the beneficial effects of 2′-FL supplementation on the microbiota-gut-brain axis and the diet-induced obese phenotype in high fat (HF)-fed mice. Male C57/BL6 mice (*n* = 6/group; six weeks old) were counter-balanced into six weight-matched groups and fed either a low-fat (LF; 10% kcal as fat), HF (45% kcal as fat) or HF diet with 2′-FL (HF_2′-FL) at 1, 2, 5 and 10% (*w*/*v*) in drinking water for six weeks. General phenotypes (body weight, energy intake, fat and lean mass), cecal microbiome and metabolites, gut-brain signaling, intestinal permeability and inflammatory and lipid profiles were assessed. Only 10% 2′-FL, but not 1, 2 or 5%, decreased HF diet-induced increases in energy intake, fat mass and body weight gain. A supplementation of 10% 2′-FL changed the composition of cecal microbiota and metabolites compared to LF- and HF-fed mice with an increase in *Parabacteroides* abundance and lactate and pyruvate, respectively, whose metabolic effects corresponded to our study findings. In particular, 10% 2′-FL significantly reversed the HF diet-induced impairment of cholecystokinin-induced inhibition of food intake. Gene expressions of interleukin (IL)-1β, IL-6, and macrophage chemoattractant protein-1 in the cecum were significantly downregulated by 10% 2′-FL compared to the HF group. Furthermore, 10% 2′-FL suppressed HF diet-induced upregulation of hepatic peroxisome proliferator-activated receptor gamma, a transcription factor for adipogenesis, at the gene level. In conclusion, 10% 2′-FL led to compositional changes in gut microbiota and metabolites associated with improvements in metabolic profiles and gut-brain signaling in HF-fed mice. These findings support the use of 2′-FL for modulating the hyperphagic response to HF diets and improving the microbiota-gut-brain axis.

## 1. Introduction

Obesity is defined by the World Health Organization as “abnormal or excessive fat accumulation that presents a risk to health” (http://www.who.int/topics/obesity/en/) [1]. Obesity has reached epidemic proportions worldwide [2] and is a substantial risk factor for metabolic complications such as dyslipidemia, abdominal obesity, insulin resistance, and hypertension [3].

Accumulating evidence suggests the gut microbiota plays a crucial role in the development of obesity [3,4]. Under normal physiological conditions, the gut microbiota has symbiotic interactions with the host, conferring beneficial effects on host physiology and behavior [5]. However, compositional and functional alterations of the gut microbiota, i.e., dysbiosis, induced by lifestyle factors have been implicated in the development and progression of metabolic disturbances including obesity as well as other pleotropic physiologic effects [6]. In particular, high fat (HF) diet-induced obesity has been characterized by a decrease in overall microbiome diversity [7], an increase in the *Firmicutes* to *Bacteroidetes* ratio [8] and production of pro-inflammatory bacterial products such as lipopolysaccharides (LPS) [9]. Interestingly, conventionalization of germ-free animals with fecal microbiota from obese donors resulted in recapitulation of the donor phenotype [10], identifying the gut microbiota as a potential driver of obesity. HF-induced gut dysbiosis also has been linked to chronic low-grade intestinal inflammation and impaired gut barrier integrity [11,12], allowing translocation of LPS from the lumen into the circulation, resulting in low-grade or metabolic endotoxemia [13]. Studies have demonstrated that chronic low-dose LPS administration can trigger macrophage infiltration and inflammation of adipose tissue, fat accumulation in hepatic and visceral fat tissue, and insulin resistance [14,15]. Circulating LPS also can induce hyperphagia and weight gain by inhibiting vagal afferent-mediated intestinal satiety signaling [14].

The gut microbiota can influence feeding behavior via communication with the gut-brain axis [15]. The gut–brain axis is a bidirectional communication system between the gastrointestinal (GI) tract and the brain. It monitors the presence or absence of nutrients and triggers appropriate physiological and behavioral changes, including the regulation of food intake and reward behavior [16,17]. For example, in the presence of food, cholecystokinin (CCK) as one of the anorexigenic gut hormones is released from its enteroendocrine cells (I-cells), binds to CCK-1 receptors on vagal afferent neurons, and activates second order neurons in the nucleus of solitary tract (NTS) and area postrema (AP) of the brainstem, ultimately suppressing food intake [18]. However, this vagally-mediated gut signaling is impaired with HF feeding [19]. In particular, HF diets significantly decrease the expression of CCK-induced c-Fos in the NTS, a marker of neuronal activation [20]. In particular, HF diet-induced alterations in the gut microbiota promote gut barrier dysfunctions, thus increasing circulating LPS levels, which can inhibit CCK-induced intestinal signaling by altering the function of vagal afferent neurons, leading to hyperphagia and body weight gain [15,19,21].

With the critical roles of the gut microbiota and its communication with the gut-brain axis in the pathogenesis of obesity and metabolic complications, modulation of the gut microbiota composition has been an attractive target to prevent and/or treat those abnormalities [22,23,24]. In particular, since the diet has been associated with critical alterations in the gut microbiota [25], the use of non-invasive dietary approaches such as prebiotics in the presence of HF challenge have been reported to increase beneficial bacteria abundance, decrease local (gut, adipose) and systemic inflammation and preserve vagally-mediated satiety signaling, which is all associated with suppression of adiposity and weight gain [23,26,27]. The underlying mechanisms responsible for these metabolic benefits are not only via modulation of the host gut microbiota composition but also via associated changes in the production of microbial metabolites. For examples, short-chain fatty acids generated by fermentation of soluble dietary fibers provide energy for colonocytes inhibit the growth of pathogens and control glucose metabolism via activation of intestinal gluconeogenesis [28]. Bile acid-derivatives contribute to maintaining intestinal barrier function and to regulate glucose and energy homeostasis [29]. In addition, indole derivatives are known to increase expression of anti-inflammatory genes and strengthen epithelial cell barrier properties [30].

The oligosaccharide 2′-fucosyllactose (2′-FL) is the predominant oligosaccharide found in human milk [31]. The oligosaccharide 2′-FL primarily acts as a prebiotic and its beneficial effects have been documented in preclinical and clinical studies [32,33,34,35]. One of the well-studied functions is its bifidogenic effect. In particular, *Bifidobacterium longum subsp. infantis* and *Bifidobacterium bifidum* have shown to grow well in 2′-FL containing culture media [35]. The oligosaccharide 2′-FL also exerts anti-inflammatory and immune-modulating effects. Grabinger et al. reported a colitis-protective effect by oral 2′-FL administration in Il10^-^/^-^ mice demonstrated by downregulated expression of inflammatory markers and increased epithelial integrity; this effect was associated with the expansion of the *Ruminococcus gnavus* [33]. It has been also shown in mice that 2′-FL can protect against necrotizing enterocolitis by restoring mesenteric perfusion via upregulation of eNOS expression [36]. Furthermore, 2′-FL has been associated with improved cognitive functions measured by behavior tests and hippocampal long-term potentiation in rats. Notably, the vagally mediated gut-brain axis was involved in this 2′-FL-mediated cognitive benefits [32].

Despite increasing evidence on the promising aspects of 2′-FL, there is limited knowledge on the potential benefits of 2′-FL in obesity. Thus, the aim of this study was to investigate the protective effects of 2′-FL against HF diet-induced obesity and its comorbidities in mice. We hypothesized that 2′-FL supplementation would improve gut-brain signaling, inflammatory profiles, and hyperphagic phenotypes in association with compositional changes in the gut microbiota and metabolites in HF diet-induced obese mice. We also sought to identify the optimal dose for 2′-FL supplementation for the prevention of HF diet-induced metabolic abnormalities. To achieve these aims, we fed mice a HF diet supplemented with 2′-FL at 1, 2, 5 and 10% (*w*/*v*) in drinking water for six weeks and investigated changes in general phenotypes (body weight, energy intake, fat and lean mass), intestinal permeability, inflammatory and lipid profiles, gut-brain signaling and cecal microbiome and metabolites. The range of 2′-FL supplementation was determined based on the no-observable-adverse-effect level (NOAEL) of 5000 mg/kg body weight/day for rodents [37].

## 2. Methods and Materials

### 2.1. Animals and Diets

Animals were maintained and handled in accordance with protocols approved by the Institutional Animal Care and Use Committee (University of California, Davis, CA, USA). Male C57/BL6 mice (*n* = 6/group; 6 week old, JAX, Sacramento, CA, USA) were split into six weight-matched groups and fed ad libitum either a low-fat (LF; 10% kcal as fat; D12450J; Research Diets, New Brunswick, NJ, USA), high-fat (HF; 45% kcal as fat; D08091803B; Research Diets) or HF diet with 2′-FL (HF_2′-FL; with 2′-FL of 98.4% purity provided by BASF, Ludwigshafen, Germany; batch no. 012644-L 04) at 1, 2, 5 or 10% (*w*/*v*) in drinking water for 6 weeks (Figure S1, Table S1). All animals were housed individually at 22 °C with 12 h: 12 h light-dark cycles with ad libitum access to food and water except when being fasted as described. Body weight and food and water intake were measured twice a week. Food efficiency was determined as weight gain (g)/energy intake (kcal). Body composition for fat and lean mass was analyzed in live animals using EchoMRI-100 TM from Echo Medical Systems (Houston, TX, USA).

In the present study, HF diet-induced obesity was defined as described in [38] where the degree of obesity is expressed by comparing the body weight (or fat) of the high-fat fed group with control animals ingesting a LF diet; an increase in body weight of 10–25% is defined as moderate obesity. This is similar to that previously reported by our group [12,24,26].

After 6 weeks of respective diets, mice were fasted for 4 h, ip injected with CCK (20 µg/kg) to determine c-Fos expression in hindbrain, and euthanized using deep anesthesia induced with pentobarbital (Fatal Plus, Vortech Pharmaceuticals, Dearborn, MI, USA; 300 mg/kg; i.p.). Blood was collected via cardiocentesis in K3-EDTA tubes and centrifuged at 1000× *g* for 10 min at 4 °C for plasma collection. The liver, ileum, cecum and contents, colon, epididymal fat pads, nodose ganglion, and brain were collected. Plasma and all of the tissues were snap-frozen and stored −80 °C until analysis.

### 2.2. CCK Sensitivity Assessment

After 4 weeks on their respective diets, mouse sensitivity to the satiating effect of CCK was tested. Experiments were performed at the onset of the dark phase. Mice were fasted on wire-bottom cages for 6 h during the light phase. At the onset of the dark phase, CCK (octapeptide, sulfated, Bachem, Torrance, CA, USA, 100 µL at 3 µg/kg; i.p.) or saline (100 µL; i.p.) was administered. Individual food was placed in the cage and food intake was recorded at every 20 min for one hour.

### 2.3. Oral Glucose Tolerance Test (OGTT)

After 5 weeks, mice were fasted for 6 h and orally gavaged with a glucose solution (2 g/kg body weight using 50% dextrose solution (Dextrose Injection, USP, Hospira. Lake Forest, IL, USA). Glycemia was measured by using a glucometer (Accu-Chek Performa, Roche, Mannheim, Germany) before (0 min) and after (15, 30, 60, 90, and 120 min) glucose administration.

### 2.4. RNA Extraction and Quantitative RT-PCR

Total RNA from ileum and colon samples was extracted using the TRIzol reagent (Life Technologies, Grand Island, NY, USA). cDNAs were synthesized from 1 μg of purified RNA samples using iScript cDNA synthesis kit (Bio-Rad, Hercules, CA, USA) following the manufacturer’s protocol. Real-time PCR was performed with the QuantStudio 6 Flex Real-Time PCR system (Thermo Fisher Scientific, Waltham, MA, USA) using SyberGreen master mix (Life Technologies) for detection. GAPDH was used as a housekeeping gene. Genes of interest were analyzed according to the 2^−ΔΔCT^ method [39] and compared with control samples. Primer sequences are provided in Table S2.

### 2.5. Immunofluorescence

Hindbrains were cryosectioned at 30 μm thickness and stained for c-Fos protein expression in the NTS and AC regions. Sections were permeabilized in PBST (phosphate-buffered saline containing 0.1% Tween 20, Sigma–Aldrich), blocked in 5% normal goat serum in 0.2% Triton X-100 (Sigma-Aldrich) in PBST for 1 h at room temperature, and incubated overnight in a primary antibody (c-Fos at 1:100; Cell Signaling Technology, Beverly, MA, USA) at 4 °C. After washes, signals were revealed by incubation with a secondary antibody (1:500; Alexa Fluor 647, Invitrogen, Carlsbad, CA, USA) in blocking buffer for 1 h in the dark at room temperature. For visualization of vagal afferents, sections were incubated with isolectin GS-IB4 Alexa Fluor 594 (IB4, 1:500; Molecular Probes, Eugene, OR, USA). Nuclei were counterstained with 4′,6-diamidino-2-phenylindole (DAPI, 1:5,000; Invitrogen) for 5 min followed by washes. Sections mounted on slides were closed with Prolong antifade mounting medium (Molecular Probes). Images were acquired using a confocal microscope (Leica TCS SP8 STED 3X; Leica, Wetzlar, Germany) and quantified in a blinded manner using Imaris Software (Bitplane, Zurich, Switzerland).

### 2.6. Histology

For hematoxylin and eosin (H and E) staining, epididymal adipose tissues were fixed in 10% neutral-buffered formalin (Thermo Fisher Scientific, Waltham, MA, USA) overnight and transferred to 70% ethanol for one day. Afterwards, the tissues were processed in a routine manner for paraffin sections (Tissue Tek VIP Tissue Processor; Sakura Finetek USA, Torrance, CA, USA). Paraffin-embedded sections (5 μm) were cut and stained with H and E (Sigma-Aldrich) for microscopic examination (Olympus BX60, Waltham, MA, USA) at 20× magnification. To quantitate adipocyte size, the H and E-stained sections were analyzed using the ImageJ software (National Institutes of Health, Bethesda, MD, USA).

### 2.7. Hepatic Lipid Accumulation

Hepatic lipid accumulation was qualitatively assessed by Oil Red O (ORO) staining and hematoxylin as a counter nuclear stain. Briefly, frozen sections (12 μm) were post-fixed with 4% PFA and stained with 0.37% Oil Red O in 60% of isopropanol for 15 min and washed three times with PBST. Sections were examined under a light microscope (Olympus BX60) at 40× magnification. The ORO-positive pixels were determined using ImageJ software. The hepatic triglyceride (TG) was quantitatively determined using a commercial kit (Fisher Diagnostics, Middletown, VA, USA) following the manufacturer’s protocol. TG concentration was expressed relative to wet liver weight (mg/g).

### 2.8. Blood Analysis

Plasma levels of LPS-binding protein (LBP; Hycult Biotech, Uden, The Netherlands) and lipocalin-2 (Lcn-2; R&D Systems, Minneapolis, MN, USA) levels were detected in plasma via enzyme-linked immunosorbent assay (ELISA) according to the manufacturers’ protocol.

### 2.9. Microbiota DNA Sequencing

Genomic DNA from cecal samples was extracted using Zymobiomics DNA Miniprep Kit (Zymo Research, Irvine, CA, USA). The V4 region of the 16S RNA gene was amplified in triplicate with barcoded PCR primers F515 and R806 as previously described [40]. Amplicons were verified by gel electrophoresis, combined, purified, and sent to the UC Davis Genome Center for library preparation and high throughput 250-bp paired-end sequencing using the Illumina MiSeq platform. Resulting raw data was demultiplexed with sabre [41] and then imported into QIIME2-2019.7 [42]. Bases before base pair 21 and after base pair 242 for the forward read, before base pair 20 and after base pair 250 for reverse read were trimmed. Trimmed reads were processed with DADA2 [43]. Trimmed and filtered sequences were aligned and taxonomy was assigned using the 99% SILVA naïve Bayeian classifier in QIIME 2 v2019.7 [41]. Bacterial abundances were determined at all phylogenic levels and normalized by log transformation.

### 2.10. Metabolomic Analysis

20 µL of supernatant from each cecum sample and standard pool dilution were reacted with 20 µL of 200 mM N-(3-Dimethylaminopropyl)-N’-ethylcarbodiimide hydrochloride in 5% pyridine and 40 µL of 100 mM 2-nitrophenylhydrazine in 80% ACN/H_2_O (*v*/*v*) with 50 mM HCl. Mixtures were incubated for 30 min at 40 °C before adding 400 µL of 10% ACN/H_2_O (*v*/*v*) to each sample. Samples were centrifuged and transferred into a 96-well injection plate for triple quadrupole LC-MS/MS analysis (using Agilent 6490 triple quadrupole mass spectrometer equipped with Agilent 1290 infinity LC system, and an Agilent InfinityLab Poroshell 120 EC-C18, 2.1 × 100 mm, 1.9 µm column). The samples were detected in positive mode using a dynamic multiple reaction monitoring MRM method.

### 2.11. Statistical Analysis

Unless stated otherwise (microbiome analysis), statistical analysis was performed using Prism software (Prism 8.1.2; GraphPad Software, La Jolla, CA, USA). The ROUT test was used to identify and exclude outliers. Two-factor repeated-measures analysis of variance (ANOVA) was used to analyze body weight and body composition and one-factor ANOVA was performed to analyze data from energy intake, histology, immunofluorescence, PCR and biochemical analyses (Table S3). Paired Student’s t test was used to determine statistical significance within groups (for CCK sensitivity). Correlations between cecal microbiome abundance and metabolites and between LBP and Lcn-2 were determined by using the nonparametric Spearman and parametric Pearson correlation, respectively. Differences between groups were analyzed by using Tukey’s post hoc tests. Differences were considered significant if *p* < 0.05. Data are presented as means ± SEMs. The linear discriminant analysis (LDA) effect size (LEfSe) method was used to identify taxa that were significantly differentially abundant for each group [44]. The METAGENassist platform [45] was used for multivariate statistical analysis. Differences in abundances among groups were assessed using Kruskal–Wallis test with Dunn’s post hoc test.

## 3. Results

### 3.1. Supplementation of 2′-FL Improves Hyperphagic Phenotypes

**Figure 1:**
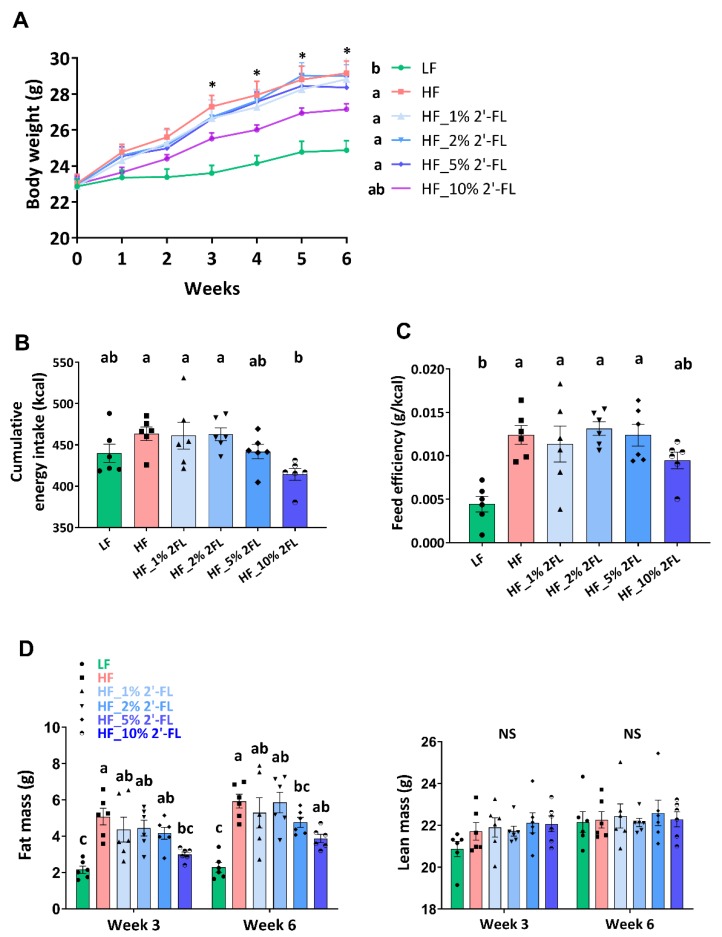
Effect of 2′-FL supplementation on hyperphagic phenotypes. Body weight (**A**), cumulative energy intake (for 5 weeks; (**B**)), feed efficiency (weight gain (g)/energy intake (kcal) for 5 weeks; (**C**)), and body composition (**D**) in mice fed an LF, HF, or HF_2′-FL (1, 2, 5 or 10%) diet for six weeks. Two-factor repeated-measures ANOVA was used to analyze body weight and body composition. One-factor ANOVA was performed to analyze data from energy intake and food efficiency. Differences between groups were analyzed by using Tukey’s post hoc tests. Values are means ± SEMs, *n* = 6/group. Histogram with different letters (a or b) denotes mean values that are statistically different at *p* < 0.05; for all variables with the same letter, the difference between the means is not statistically significant and if two variables have different letters, they are significantly different. NS, not significant. * denote significant differences among groups at *p* < 0.05. ANOVA, analysis of variance; HF, high fat; HF_x% 2′-FL, HF with x% 2′-FL (*w*/*v*) in drinking water; LF, low fat; 2′-FL, 2-fucosyllactose.

After the third week of diet treatment, HF feeding significantly increased body weight compared to the LF group (17%; LF vs. HF, *p* < 0.001, Figure 1A). Mice fed a HF diet supplemented with 10% 2′-FL gained significantly less weight than mice fed a HF diet alone. Though not statistically significant (*p* = 0.09), HF_10% 2′-FL fed mice did gain more weight than mice fed a LF diet. Supplementation of HF-fed mice with 2′-FL at 1, 2 or 5% had no significant effect on body weight (HF vs. HF_1, 2, or 5% 2′-FL, *p* > 0.05). Cumulative food intake and feed efficiency were calculated based on the first five weeks of the study. The HF group showed higher cumulative energy intake compared to the LF group but this did not reach statistical significance (LF vs. HF, *p* > 0.05; Figure 1B). However, 10% 2′-FL supplementation decreased food intake compared to HF fed mice (HF_10% 2′-FL vs. HF, *p* < 0.05). HF feeding led to a significant increase in feed efficiency (LF vs. HF, *p* < 0.01; Figure 1C), which was partially suppressed by 10% 2′-FL supplementation although this did not reach statistical significance (LF vs. HF_10% 2′-FL, *p* > 0.05, Figure 1C). Fat and lean mass were measured at the third and sixth week of the experimental feeding (Figure 1D). There was no difference in lean mass among groups. However, HF feeding significantly increased fat mass compared to the LF group (LF vs. HF, *p* < 0.001) and this increase was suppressed by 10% 2′-FL supplementation (HF vs. HF_10% 2′-FL, *p* < 0.01). Supplementation of HF diet with 1, 2 or 5% 2-FL had no significant effect on cumulative food intake, feed efficiency or fat mass (cumulative food intake: LF or HF vs. HF_1, 2, or 5% 2′-FL, *p* > 0.05; feed efficiency: LF vs. HF_1% 2′-FL, *p* < 0.01; vs. HF_2% 2′-FL, *p* < 0.001; vs. HF_5% 2′-FL, *p* < 0.01; fat mass: LF vs. HF_1% 2′-FL, *p* < 0.01; vs. HF_2% 2′-FL, *p* = 0.0001; vs. HF_5% 2′-FL, *p* < 0.05). No significant effect was observed by HF diet on plasma levels of glucose (area under the curve) compared to the LF group (LF: 24426 ± 4791 vs. HF: 32906 ± 5360 mg/dL × 120 min, *p* > 0.05; data not shown).

### 3.2. Supplementation of 10% 2′-FL Preserves the Integrity of Vagally-Mediated Gut-Brain Signaling

**Figure 2:**
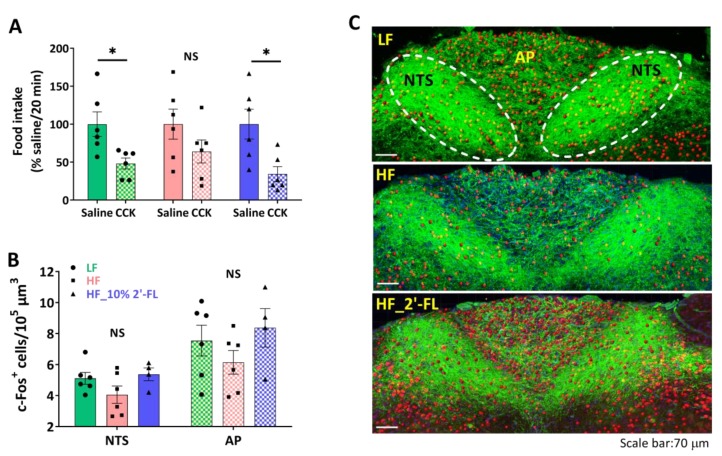
Effect of 10% 2′-FL supplementation on vagally-mediated gut-brain signaling. CCK sensitivity (**A**) and c-Fos positive cells in the nucleus of the solitary tract (NTS) and area postrema (AP) in the hindbrain (**B**,**C**; blue: DAPI, green: ib4, red: c-Fos) in mice fed an LF, HF, or HF_10% 2′-FL diet for six weeks. Paired Student’s t-test was used to determine statistical significance within groups for CCK sensitivity. One-factor ANOVA was performed to analyze c-Fos expression in the NTS and AP areas. Differences between groups were analyzed by using Tukey’s post hoc tests. Values are means ± SEMs, *n* = 6/group. * denotes significant differences in food intake between saline and CCK treatment at *p* < 0.05. NS, not significant. ANOVA, analysis of variance; CCK, cholecystokinin; DAPI, 4′,6-diamidino-2-phenylindole; HF, high fat; HF_10 % 2′-FL, HF with 10% 2′-FL (*w*/*v*) in drinking water; IB4, isolectin B4; LF, low fat; 2′-FL, 2-fucosyllactose.

To investigate the effect of 2′-FL supplementation on intestinal satiety signaling, we determined the ability of exogenous administration of CCK to decrease food intake (Figure 2A). The administration of CCK in LF-fed mice significantly decreased food intake compared to the saline control (CCK vs. saline, *p* < 0.05). In contrast, there was no significant effect of CCK on food intake in mice fed a HF diet (CCK vs. saline, *p* > 0.05). A supplementation of 10% 2′-FL restored the CCK-induced inhibition of food intake (CCK vs. saline, *p* < 0.05).

To investigate the ability of CCK to activate second order neurons in the brainstem, CCK was administered two hours before euthanasia and hindbrain tissues were immunostained to determine c-Fos expression, a measure of neuronal activation, in the NTS and AP, regions where vagal afferents terminate (Figure 2B,C). There was little difference among groups in the number of c-Fos-positive cells in both NTS and AP areas.

### 3.3. Compositional Changes in the Gut Microbiota and Metabolites by 10% 2′-FL Supplementation

**Figure 3:**
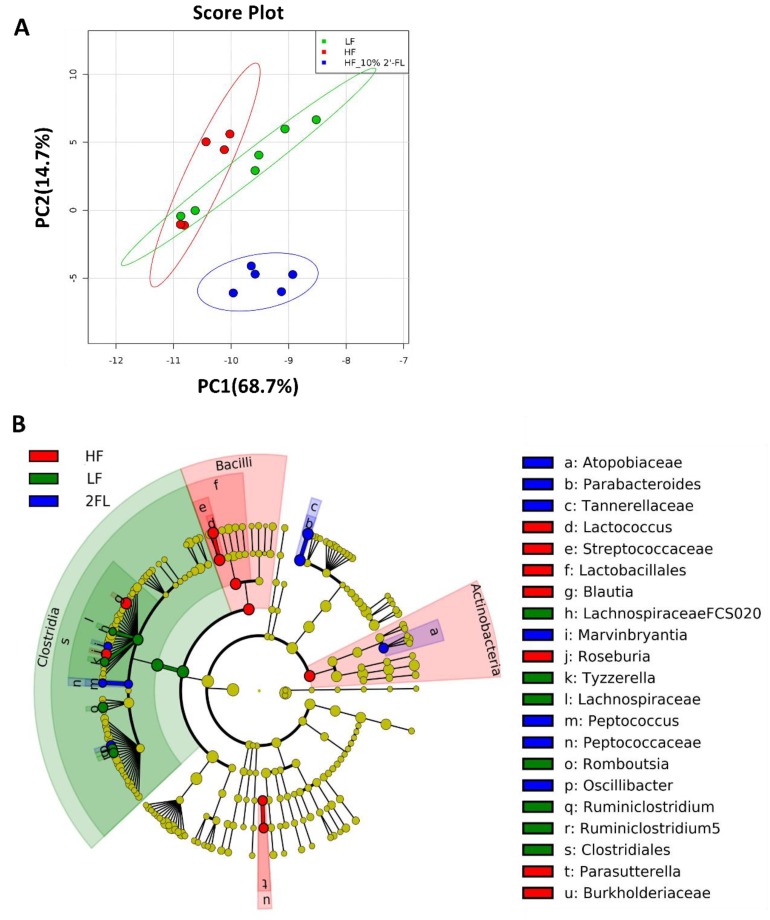
Effect of 10% 2′-FL supplementation on the composition of the gut microbiota. Principal components analysis on all taxonomic levels (**A**) of mice fed an LF, HF, or HF_10% 2′-FL diet for six weeks. The METAGENassist platform was used for multivariate statistical analysis. The linear discriminant analysis (LDA) effect size (LEfSe) method was used to identify taxa that were significantly differentially abundant for each group. Differences in abundances among groups were assessed using Kruskal–Wallis test with Dunn’s post hoc test. *n* = 5~6/group. Cladogram (**B**) generated from LEfSe analysis shows the most differentially abundant taxa enriched in microbiota; blue indicating LF, green HF, red HF_2′-FL, and yellow indicating non-significance. The six rings of the cladogram stand for domain (innermost), phylum, class, order, family and genus. HF, high fat; HF_10% 2′-FL, HF with 10% 2′-FL (*w*/*v*) in drinking water; LEfSe, linear discriminant analysis effect size; LF, low fat; 2′-FL, 2-fucosyllactose.

Gut microbiota was analyzed and described at all phylogenic levels when abundance was >0.1% (Figure 3). At all taxonomic levels, 10% 2′-FL supplementation resulted in a uniquely different gut microbiota compared to both HF and LF fed mice (Figure 3A, Figure S2). The gut microbiota composition of HF_10% 2′-FL mice was primarily characterized by the presence of *Tannerellaceae*, *Parabacteroides* and *Parabacteroides goldsteinii* at the family, genus and species level, respectively (*p* < 0.05), which belong to *Bacteroidales* (order), *Bacteroidia* (class) and *Bacteroidetes* (Phylum; Figure 3B, Figure S3). In particular, at the genus level, 10% 2′-FL supplementation also differentially increased abundance of *Peptococcus* (*p* < 0.01), *Atopobiaceae* (*p* < 0.001), *Oscillibacter* (*p* < 0.001) and *Marvinbryantia* (*p* < 0.01). On the other hand, *Blautia* (*p* < 0.001), *Lactococcus* (*p* < 0.01), *Roseburia* (*p* < 0.001) and *Parasutterella* (*p* < 0.05) were the most differentially abundant taxonomic groups in the HF group.

**Figure 4:**
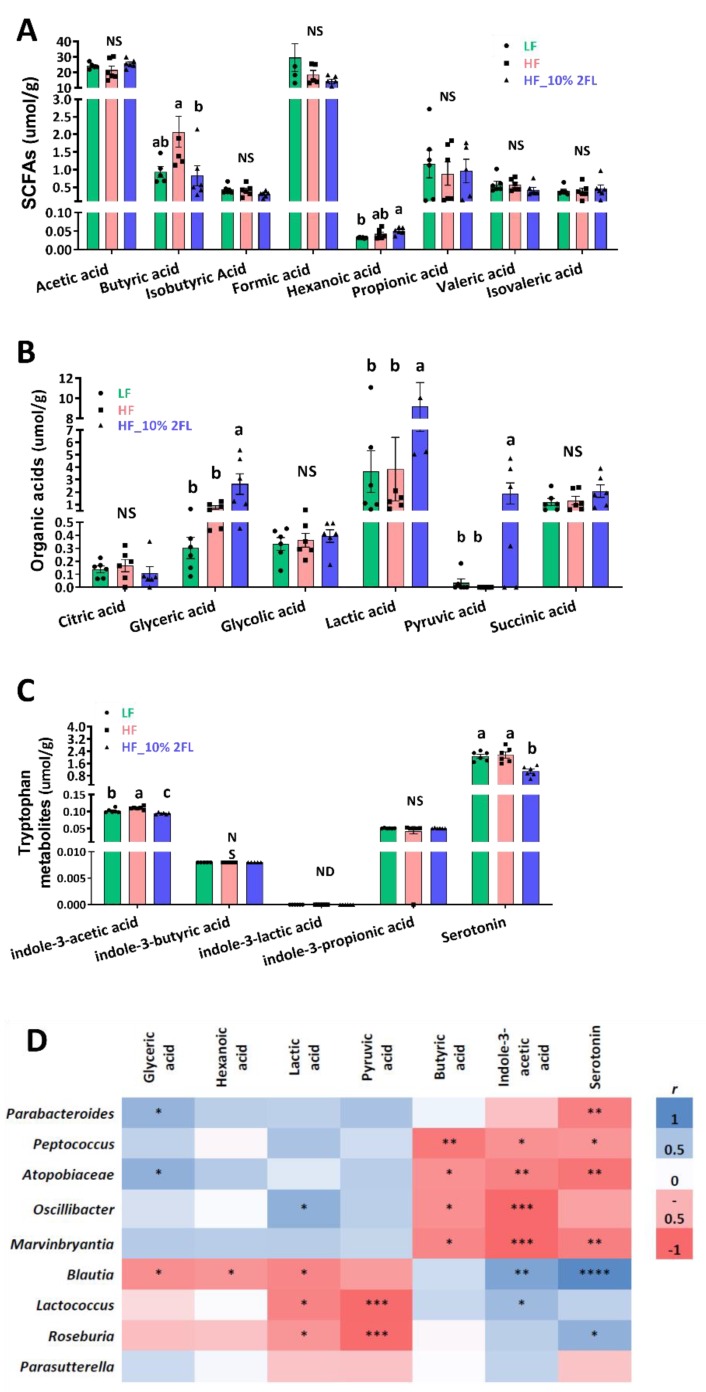
Effect of 10% 2′-FL supplementation on metabolomic profiles in the cecum of mice fed an LF, HF, or HF_10% 2′-FL diet for six weeks. One-factor ANOVA was performed to analyze metabolomic data. Differences between groups were analyzed by using Tukey’s post hoc tests. Correlation between cecal microbiome and metabolites was determined by using the nonparametric Spearman correlation analysis. Values are means ± SEMs, *n* = 6/group. Different letters denote significant differences between groups at *p* < 0.05. Metabolite concentrations (**A**–**C**) and heatmap visualization of microbiota–metabolome correlation analysis (**D**); blue and red colors indicate a positive or negative Spearman correlation, respectively. Histogram with different letters (a or b) denotes mean values that are statistically different at *p* < 0.05; for all variables with the same letter, the difference between the means is not statistically significant and if two variables have different letters, they are significantly different. ND, not detected; NS, not significant. * *p* < 0.05, ** *p* < 0.01, *** *p* < 0.001, **** *p* < 0.0001. ANOVA, analysis of variance; HF, high fat; HF_10 % 2′- FL, HF with 10% 2′-FL (*w*/*v*) in drinking water; LF, low fat; SCFA, short chain fatty acid; 2′-FL,2-fucosyllactose.

A supplementation of 10% 2′-FL was associated with significant shifts in metabolites of the cecum (Figure 4A–C). Concentrations of glyceric, lactic, hexanoic and pyruvic acid were significantly elevated by 10% 2′-FL supplementation compared to LF and/or HF groups (glyceric acid: HF_10% 2′-FL vs. LF or HF, *p* < 0.05; hexanoic acid: HF_10% 2′-FL vs. LF, *p* < 0.01); lactic acid: HF_10% 2′-FL vs. LF, *p* < 0.05; vs. HF, *p* < 0.01; pyruvic acid: HF_10% 2′-FL vs. LF or HF, *p* < 0.05). Concentrations of butyric and indole-3-acetic acid and serotonin were significantly decreased by 10% 2′-FL supplementation compared to the LF group (butyric acid; HF_10% 2′-FL vs. HF, *p* < 0.05) or both LF and HF groups (indole-3-acetic acid: HF_10% 2′-FL vs. LF, *p* < 0.05; vs HF, *p* < 0.0001; serotonin; HF_10% 2′-FL vs. LF, *p* < 0.01; vs HF, *p* < 0.001).

Correlation analysis showed significantly positive and/or negative correlations between microbiome and metabolomes in the cecum (Figure 4D). In particular, glyceric acid was positively correlated with *Parabacteroides* (*r* = 0.58, *p* < 0.05) and *Atopobiaceae* (*r* = 0.61, *p* < 0.05) and negatively correlated with *Blautia* (*r* = −0.56, *p* < 0.05). Lactic acid was positively correlated with *Oscillibacter* (*r* = 0.61, *p* < 0.05) and negatively correlated with *Blautia* (*r* = −0.61, *p* < 0.05)*, Lactococcus* (*r* = −0.7, *p* < 0.01) and *Roseburia* (*r* = −0.55, *p* < 0.05). Indole-3-acetic acid was positively correlated with *Blautia* (*r* = 0.69, *p* < 0.01) and *Lactococcus* (*r* = 0.55, *p* < 0.05) and negatively correlated with *Peptococcus* (*r* = −0.55, *p* < 0.05), *Atopobiaceae* (*r* = −0.63, *p* < 0.01), *Oscillibacter* (*r* = −0.79 *p* < 0.001) and *Marvinbryantia* (*r* = −0.79 *p* < 0.001). Serotonin was positively correlated with *Blautia* (*r* = 0.85, *p* < 0.0001) and *Roseburia* (*r* = 0.61, *p* < 0.05) and negatively correlated with *Parabacteroides* (*r* = −0.65, *p* < 0.01)*, Peptococcus* (*r* = −0.52, *p* < 0.05)*, Atopobiaceae* (*r* = −0.72, *p* < 0.01)*, Marvinbryantia* (*r* = −0.65, *p* < 0.01). Furthermore, there were negative correlations of hexanoic acid with *Blautia* (*r* = -0.52, *p* < 0.05), pyruvic acid with *Lactococcus* (*r* = −0.78, *p* < 0.001) and *Roseburia* (*r* = −0.77, *p* < 0.001), and butyric acid with with *Peptococcus* (*r* = −0.7, *p* < 0.01)*, Atopobiaceae* (*r* = −0.55, *p* < 0.05)*, Oscillibacter* (*r* = −0.55, *p* < 0.05) and *Marvinbryantia* (*r* = −0.61, *p* < 0.05).

### 3.4. Supplementation of 10% 2′-FL Attenuates HF-Induced Inflammation at the Local and Systemic Levels

**Figure 5:**
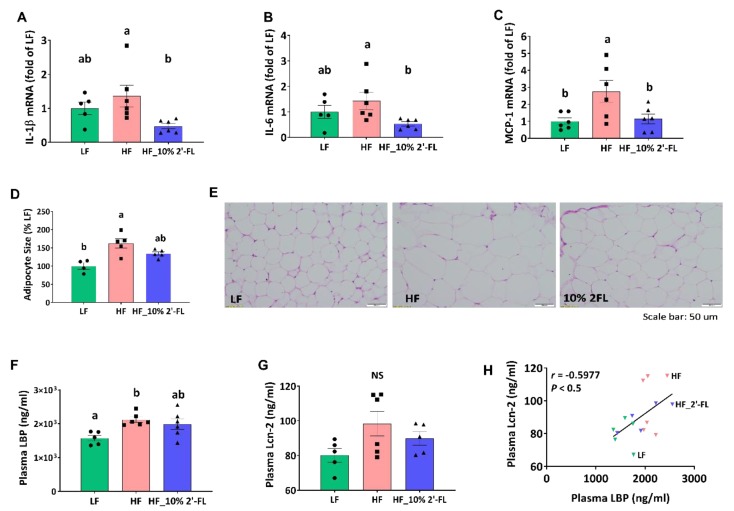
Effect of 10% 2′-FL supplementation on HF-induced inflammation at the local and systemic levels. Gene expression of pro-inflammatory markers in the cecum (**A**,**B**), gene expression of a macrophage infiltration marker and (**C**) and adipocyte size (**D**,**E**) in white adipose tissue, circulating LPS (**F**) and Lcn-2 (**G**), and correlation between plasma LPS and Lcn-2 (**H**) in mice fed LF, HF, or HF_10% 2′-FL diet for six weeks. One-factor ANOVA was performed to analyze data from histology, PCR and biochemical analyses. Differences between groups were analyzed by using Tukey’s post hoc tests. Correlation between LBP and Lcn-2 was determined by using the parametric Pearson correlation analysis. Values are means ± SEMs, *n* = 6/group. Histogram with different letters (a or b) denotes mean values that are statistically different at *p* < 0.05; for all variables with the same letter, the difference between the means is not statistically significant and if two variables have different letters, they are significantly different. NS, not significant. ANOVA, analysis of variance; HF, high fat; HF_10 % 2′-FL, HF with 10% 2′-FL (*w*/*v*) in drinking water; IL-1β, interleukin 1 beta; IL-6, interleukin 6, LBP, lipopolysaccharide (LPS)-binding protein; Lcn-2; lipocalin-2; LF, low fat; MCP-1; monocyte chemoattractant protein-1; 2′-FL, 2-fucosyllactose.

In the cecum, HF feeding tended to increase the expression of pro-inflammatory cytokines interleukin (IL)-1β and IL-6 compared to the LF group, although this did not reach statistical significance (Figure 5A,B). However, 10% 2′-FL supplementation resulted in a significant decrease in expression of both cytokines compared to the HF group (2′-FL vs. HF, *p* < 0.05). In epididymal adipose tissue, HF feeding significantly increased the gene expression of the pro-inflammatory marker, monocyte chemoattractant protein-1 (MCP-1; LF vs. HF, *p* < 0.05; Figure 5C) and this was significantly reduced by 10% 2′-FL supplementation (2′-FL vs. HF, *p* < 0.05). Furthermore, HF feeding significantly increased the size of adipocytes compared to the LF group (LF vs. HF, *p* < 0.01) but this significant difference was not observed between LF and HF_10% 2′-FL groups.

HF feeding significantly increased the level of circulating LBP, a proxy measure of LPS in systemic circulation, compared to the LF group, which was partially suppressed by 10% 2′-FL supplementation (LF vs. 10% 2′-FL, *p* = 0.065; Figure 5F). The plasma level of Lcn-2 as another indicator for systemic inflammation was not significantly different among groups (LF vs. HF, *p* = 0.085; Figure 5G), but was positively correlated with the level of plasma LBP (*r* = 0.5458, *p* < 0.05; Figure 5H).

### 3.5. Supplementation of 10% 2′-FL Iimproves Lipid Metabolism in the Liver

**Figure 6:**
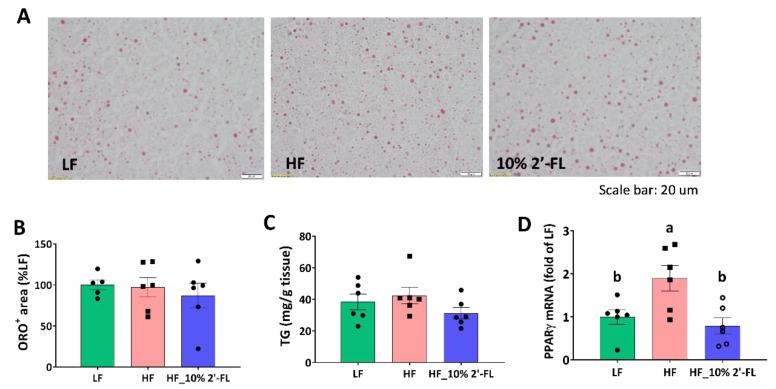
Effect of 10% 2′-FL supplementation on lipid metabolism in the liver. Lipid accumulation (**A**,**B**), triglyceride (TG, (**C**)), and gene expression of peroxisome proliferator-activated receptor gamma (PPARγ, (**D**)) in the liver of mice fed an LF, HF, or HF_10% 2′-FL diet for six weeks. One-factor ANOVA was performed for statistical analysis. Differences between groups were analyzed by using Tukey’s post hoc tests. Values are means ± SEMs, *n* = 6/group. Histogram with different letters (a or b) denotes mean values that are statistically different at *p* < 0.05; for all variables with the same letter, the difference between the means is not statistically significant and if two variables have different letters, they are significantly different. ANOVA, analysis of variance; HF_10 % 2′-FL, HF with 10% 2′-FL (*w*/*v*) in drinking water; LF, low fat; ORO, Oil Red O; 2′-FL, 2-fucosyllactose.

Based on reduced fat mass by 10% 2′-FL supplementation, lipid metabolism was further examined in the liver (Figure 6). There was no significant effect of HF diet or 2′-FL supplementation on lipid accumulation or the level of triglyceride in the liver (Figure 6A–C). However, HF feeding significantly upregulated the gene expression of peroxisome proliferator-activated receptor gamma (PPARγ), a transcription factor for adipogenesis, compared to the LF group (LF vs. HF, *p* < 0.05); this was significantly decreased by 10% 2′-FL supplementation (HF_10% 2′-FL vs. HF, *p* < 0.01; Figure 6D).

## 4. Discussion

Here, we investigated the effects of 2′-FL supplementation on the microbiota-gut-brain axis and the diet-induced obese phenotype in mice fed a HF diet. Our hypothesis was that 2′-FL supplementation would trigger compositional changes in the gut microbiota and this would be associated with improvements in gut-brain signaling, local and systemic inflammation, and hyperphagia. We also sought to determine the optimal dose for 2′-FL supplementation at 1, 2, 5 and 10% (*w*/*v*) in drinking water.

Based on improvements in the diet-induced obese phenotype (body weight, energy intake and fat mass), 10% 2′-FL was determined as the effective dose for supplementation. There was no difference in fluid intake among 2′-FL-treated groups, suggesting no adverse effects of taste by 2′-FL supplementation; fluid intake corresponded with food intake (*r* = 0.73; Figure S4). A supplementation of 10% 2′-FL led to a unique profile of the gut microbiota characterized by a differential abundance of *Parabacteroides* genus, which has been shown to have anti-obesity effects [46]. A supplementation of 10% 2′-FL also differentially increased the levels of cecal metabolites, pyruvate and lactate, which have previously been shown to decrease food intake [47,48]. The 10% 2′-FL-induced compositional changes in the gut microbiota and metabolites were associated with improvements in gut-brain signaling and metabolic profiles. A supplementation of 10% 2′-FL significantly reversed the impairment in CCK-induced inhibition of food intake. Concomitantly, 10% 2′-FL significantly decreased body weight gain, energy intake, fat mass and improved inflammatory profiles. Taken together, these data show that 10% 2′-FL supplementation led to improvements in metabolic profiles and gut-brain signaling in HF-fed mice and these improvements were associated with changes in the composition of the gut microbiota and metabolites.

Since a defining characteristic of prebiotics is “to allow specific changes in the gut microbiota both in the composition and/or activity of the gut microflora that confer benefits upon host wellbeing and health” [49], we characterized the compositional changes of the gut microbiota in mice. At all taxonomic levels, we did not observe compositional changes previously characterized in other studies, such as an increased ratio of *Firmicutes* to *Bacteroidetes* abundance by HF feeding [50]. This may be due to the highly matched composition of the semi-purified diets used in the current study, where we controlled for cellulose content as opposed to the use of standard laboratory chow [51]. The relative abundance of *Blautia* genus was greater in the HF group than LF and 2′-FL groups as previously reported [52] and it has been associated with visceral fat accumulation [53].

The supplementation of 10% 2′-FL led to a unique profile of the gut microbiota compared to LF and HF groups. Specifically, when the PCA was performed between groups (LF vs. HF, LF vs. HF_10% 2′-FL or HF vs. HF_10% 2′-FL) at all taxonomic levels, the HF_10% 2′-FL group showed a distinctly different composition of the gut microbiota compared to both LF and HF groups while some shared features were identified between LF and HF groups. Notably, the gut microbiota composition of the HF_10% 2′-FL group was characterized by differential relative abundance of the *Parabacteroides* genus, which was primarily driven by the *Parabacteroides goldsteinii* species. This species has previously been shown to have anti-obesogenic effects by enhancing gut integrity, ameliorating intestinal and systemic inflammation and improving lipid metabolism via increased adipose tissue thermogenesis [46]. Ethan et al. also reported a four-fold increase in the abundance of genus *Parabacteroides* in mice supplemented with 2′-FL after ileocecal resection [54].

The gut microbiota has the ability to generate a series of metabolites, regulating host metabolism [55], enabling them to be used as potential biomarkers of metabolic profiles such as gut inflammation [56]. In the present study, we found strong correlations between the gut microbiota and microbial metabolites. In particular, HF-induced compositional changes in microbial metabolites were positively correlated with microbiota taxa that were differentially abundant in the HF group and were negatively correlated with microbiota taxa that were characterized in the HF_10% 2′-FL group.

Serotonin is a tryptophan metabolite that can be synthesized by the host and by the gut microbiota [57]. Recent studies have shown a role for intestinal serotonin in inducing inflammation in experimental colitis, Crohn’s disease and ulcerative colitis, possibly by producing proinflammatory cytokines [58] and also by selecting for a more colitogenic microbiota [56]. However, serotonin has also been found to have anti-inflammatory actions [59], possibly by acting at the apical membrane of intestinal epithelial cell. In the current study, the gut-derived serotonin level was strongly positively correlated with *Blautia* and negatively correlated with *Parabacteroides*, the most differentially abundant genus in HF and HF_10% 2′-FL groups, respectively. A supplementation of 10% 2′-FL prevented HF-induced upregulation of pro-inflammatory cytokines (IL-1β and IL-6) in the cecum. Furthermore, these cytokines showed strong correlations with serotonin level in the cecum (*r* = 0.83 for IL-1β and *r* = 0.68 for IL-6), possibly suggesting that gut inflammation primarily originated from the compositional changes of the gut microbiota and its metabolites by HF feeding.

The 10% 2′-FL-induced compositional changes in the gut microbiota and metabolites and the resulting improvement in gut inflammation in this study may have protected intestinal barrier integrity, suppressing the translocation of bacteria-derived endotoxin LPS into the circulation as previously reported [60]. In this study, we found a significant increase in plasma LBP (as a proxy of LPS level) by HF feeding, which was partially prevented by 10% 2′-FL supplementation, indicating that 10% 2′-FL partially protected intestinal barrier permeability against HF feeding, possibly ameliorating systemic inflammation [60]. The effect of 10% 2′-FL on systemic inflammation was further supported by a positive correlation of LBP and Lcn-2 levels in plasma. Lcn-2 is a secretory glycoprotein expressed in multiple tissues and considered one of the mediators responsible for the low-grade systemic inflammation [61]. Furthermore, the increased LPS in circulation also can induce inflammation in white adipose tissue involving MCP-1 [62]. In an obese state by HF feeding, macrophages are recruited to the expanding adipose tissue and both activated macrophages and enlarged adipocytes promote the production of pro-inflammatory factors, leading to increased local inflammation [63]. In this study, HF-induced upregulation of MCP-1 as a marker of macrophage infiltration was reversed by 10% 2′-FL supplementation in visceral fat. These results confirmed the anti-inflammatory effects of 10% 2′-FL at both local and systemic levels.

In a normal physiological state, CCK is released from I cells in the duodenum in the presence of nutrients, binds to CCK-1 receptors on vagal afferents and activates neurons in the brainstem, ultimately suppressing food intake [18]. However, the chronic elevation of circulating LPS by a HF diet can alter the innervation of vagal afferent neurons, leading to impairment in CCK-induced satiety signaling [14]. Thus, we hypothesized that decreases in gut inflammation and plasma LPS by 10% 2′-FL would be associated with decreased food intake via preserving the integrity of the gut-brain signaling against HF feeding. To this end, we examined the ability of CCK to inhibit food intake in mice. As previously reported, CCK-induced inhibition of food intake was impaired by HF feeding and this was prevented by 10% 2′FL supplementation. The data suggest that preservation of the ability of intestinal satiety signals to inhibit food intake might have contributed to the decrease in food intake and improved metabolic phenotypes seen with 10% 2-FL treatment.

Another possible mechanism for 10% 2′-FL-induced decrease in food intake is through the hypophagic effects of pyruvate and lactate. Several experimental studies have found that peripherally administered pyruvate and lactate, including hepatic vein infusion, exert an inhibitory effect on food intake [47,48]. Interestingly, the suppression of food intake in response to peripheral administration of pyruvate and lactate was blocked after hepatic branch vagotomy [48], suggesting that their hypophagic effects depend on the integrity of the hepatic branch of the vagus nerve. Here, we found increased concentrations of pyruvate and lactate in the cecum in the HF_10% 2′-FL group compared HF fed mice. Pyruvate and lactate are derived from two different types of living organisms; the host and its gut microbiota. Intestinal levels of pyruvate and lactate come from ingested food and production by the gut microbiota [64]. It is likely that the increase in lactate in 10% 2′-FL-treated mice is from the gut microbiota. Gut-derived products including microbial metabolites can translocate to the liver via the portal vein, that is, the gut-liver axis [65]. Thus, it is possible that gut microbiota-derived pyruvate and lactate were transported to the liver via the hepatic portal vein, suppressing food intake in the 10% 2′-FL treated mice.

Obesity pathogenesis involves infiltration of macrophages into expanding adipose tissue and both activated macrophages and hypertrophic adipocytes contribute to local low-grade inflammation in adipose tissue and abnormal hepatic lipid metabolism [66]. Studies have previously demonstrated that animals on HF diets showed chronic inflammation in white adipose tissue and ectopic fat deposition in the liver, which was associated with increased expression of hepatic PPARγ as a transcription factor essential for adipocyte differentiation [67]. Here, we found that HF feeding caused upregulation of macrophage (MCP-1) expression in adipose tissue and adipocyte hypertrophy in white adipose tissue, which was markedly suppressed by 10% 2′-FL supplementation. It is interesting to note that in the liver, although there was no significant effect of HF diet on fat accumulation and TG levels, there was a significant upregulation of hepatic PPARγ expression by HF feeding; overexpression of PPAR-γ has shown to be sufficient in inducing hepatic fat deposition [68,69]. This increase was significantly reduced by treatment with 10% 2′-FL.

There are limitations to this study that deserve consideration. One is that we employed high-fat feeding for six weeks to induce diet-induced obesity. This protocol was sufficient to produce some characteristics at the phenotypic level (increases in body weight, food intake, and fat mass), but not at the hepatic level (no differences in ORO-positive cells and triglyceride levels). A study with duration longer than six weeks would better enable the establishment of other measures of diet-induced obesity. In addition, we showed the protective effect of 10% 2′-FL on gut barrier integrity by measuring the concentration of serum LBP as a proxy to the LPS concentration in the circulation. Future studies should use a direct assessment of intestinal tight junction permeability to further validate the role of 2′-FL in preserving the intestinal epithelial barrier.

## 5. Conclusions

In conclusion, we demonstrated that 10% 2′-FL-induced compositional changes in the gut microbiota and metabolites in HF-fed mice were associated with improvements in gut health, inflammatory profile and lipid metabolism, but also with the preserved integrity of gut-brain signaling. These compositional changes may have prevented LPS translocation and subsequent gut inflammation, consequently protecting against gut-brain axis dysfunction and progression into increase body weight, increase in adiposity and hyperphagia. Thus, these findings support the use of 2′-FL for modulating the hyperphagic response to HF diets and improving the microbiota-gut-brain axis.

## Supplementary Materials

The following are available online at https://www.mdpi.com/2072-6643/12/4/1003/s1, **Table S1.** Diet composition of LF and HF diets, **Table S2.** Primer sequences used for RT-PCR, **Table S3.** ANOVA data summary, **Figure S1.** Experimental design of the study, **Figure S2.** Effect of 10% 2′-FL supplementation on the composition of the gut microbiota, **Figure S3.** Histogram of the LDA scores from LEfSe analysis, showing the most differentially abundant taxa enriched in microbiota from mice fed an LF, HF, or 2FL (in drinking water, *w*/*v*) diet for 6 weeks, **Figure S4.** Water intake.
