# Supplementary material for: 2′-Fucosyllactose Supplementation Improves Gut-Brain Signaling and Diet-Induced Obese Phenotype and Changes the Gut Microbiota in High Fat-Fed Mice
Source: Nutrients. 2020 Apr 5;12(4):1003. doi: 10.3390/nu12041003 (PMC7231103; doi:10.3390/nu12041003)
Supplement: Supplementary file 1 [file nutrients-12-01003-s001.pdf]

**Weeks**	-1	1	2	3	4	5	6
**LF**	Acclimation	LF diet					
**HF**		HF diet					
**HF_2'-FL**		HF diet with HF_2'-FL (1, 2, 5, or 10%) in drinking water					
**Major procedures**						CCK	OGTT

Figure S1. Experimental design of the study. After 1-week acclimation, 36 male C57/BL6 mice (n = 6/group; 6 week old) were split into six weight-matched groups and fed ad libitum either a low-fat (LF; 10% kcal as fat; Research Diets D12450J), high-fat (HF; 45% kcal as fat; Research Diets D08091803B), or HF diet with 2'-FL (HF_2'-FL; with 2'-FL provided by BASF, Germany) at 1, 2, 5, or 10% (w/v) in drinking water for 6 weeks. CCK, cholecystokinin sensitivity test; HF, high fat; HF_x% 2'-FL, HF with x% 2'-FL (w/v) in drinking water; LF, low fat; OGTT, oral glucose tolerance test; 2'-FL, 2-fucosyllactose.

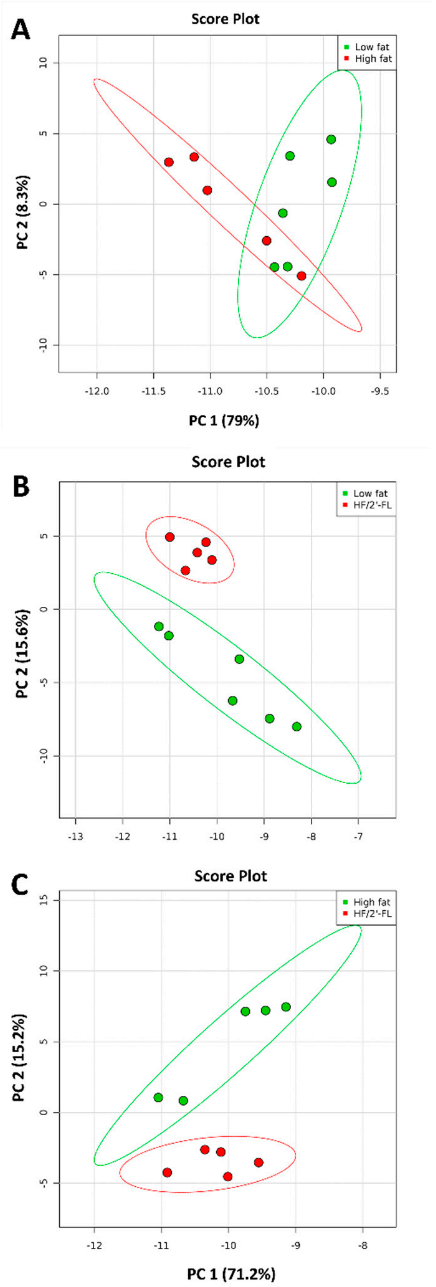

Figure S2. Effect of 10% 2'-FL supplementation on the composition of the gut microbiota. Principal components analysis on all taxonomic levels of mice fed an LF, HF, or HF/2'-FL diet for 6 weeks; between LF and HF (A), LF and 10% 2'-FL (B), and HF and 10% 2'-FL (C). he METAGENassist platform was used for multivariate statistical analysis. n = 5~6/group. HF, high fat; HF/2'-FL, HF with 10% 2'-FL (w/v) in drinking water; low fat; 2'-FL, 2-fucosyllactose.

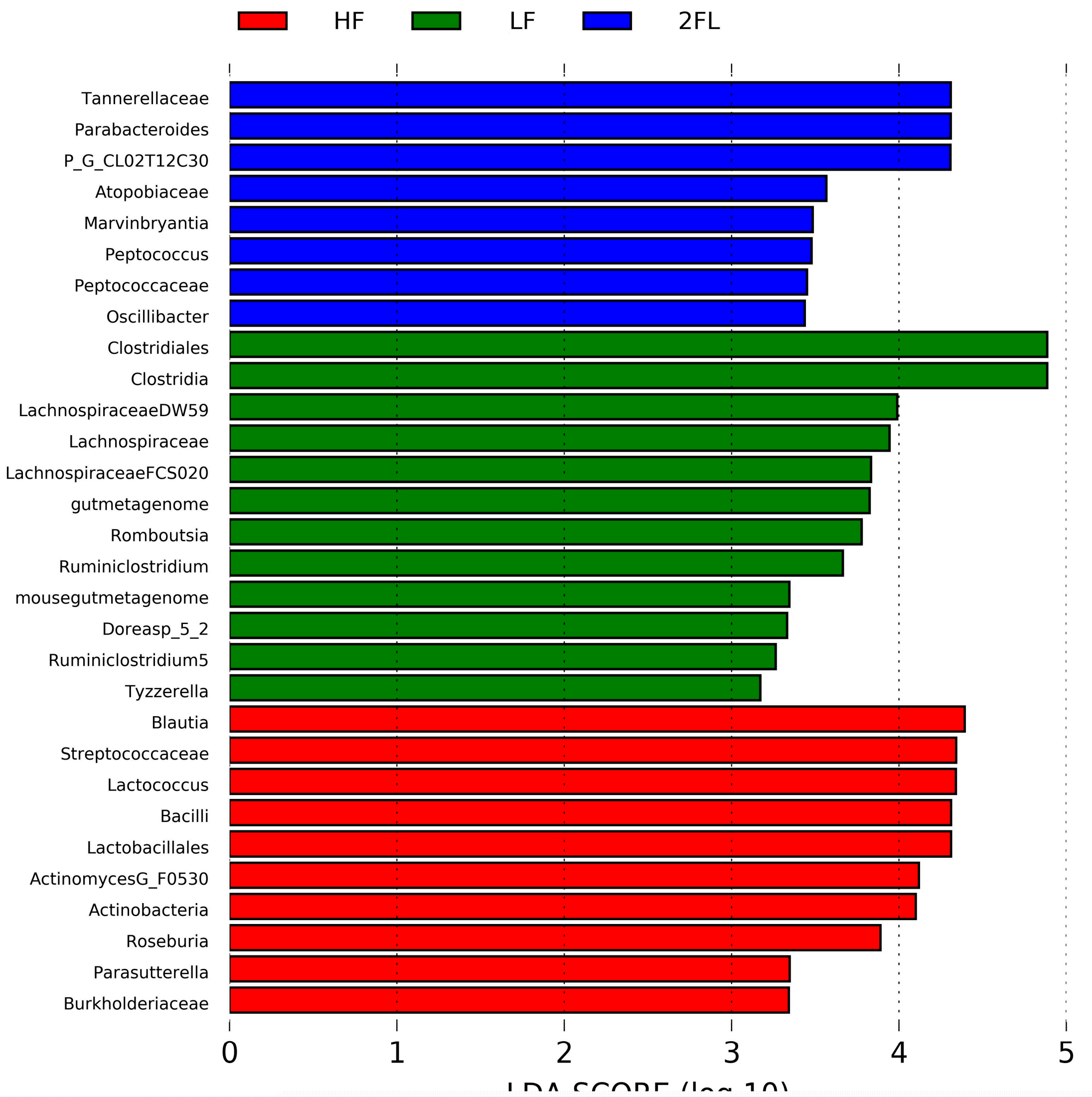

Figure S3. Histogram of the LDA scores from LEfSe analysis, showing the most differentially abundant taxa at all taxonomic levels enriched in microbiota from mice fed an LF, HF, or 2FL (in drinking water, w/v) diet for 6 weeks. n = 5~6/group. The LEfSe method was used to identify taxa that were significantly differentially abundant for each group. Differences in abundances among groups were assessed using Kruskal–Wallis test with Dunn’s post hoc test. The threshold of the logarithmic linear discriminant analysis score was 4.0. HF, high fat; 2FL, HF with 10% 2’-FL (w/v) in drinking water; LEfSe, linear discriminant analysis effect size; LF, low fat; P_G_, *Parabacteroides goldsteinii*;.

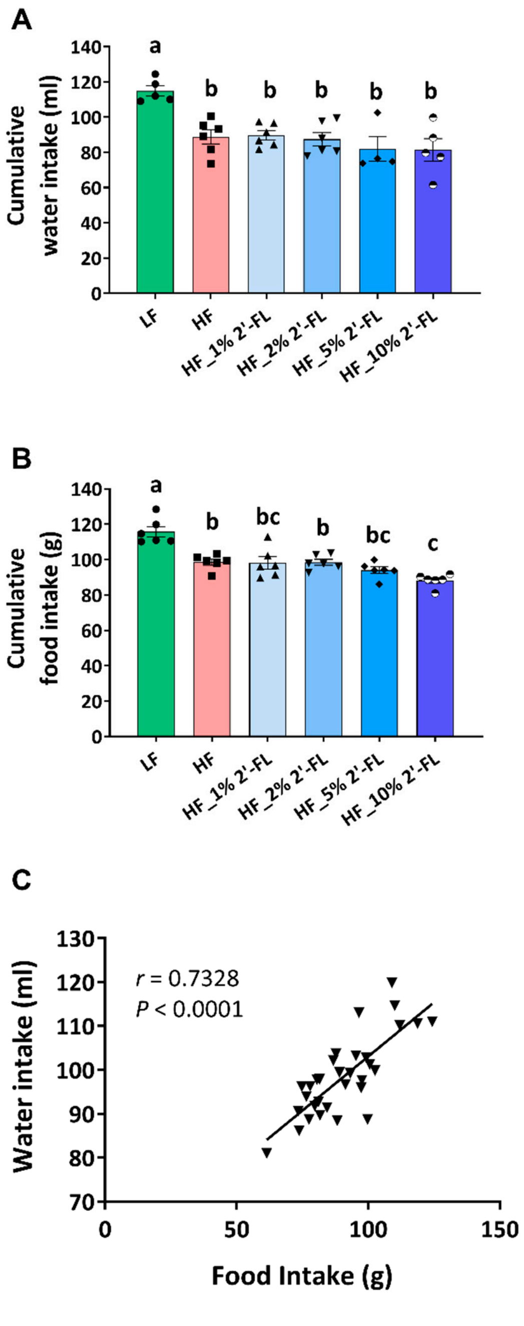

Figure S4. Water intake**.** Cumulative water (A) and food (B) consumption and the correlation between water and food intake (C) in mice fed an HF_2'-FL (1, 2, 5, or 10% (w/v) in drinking water) diet for 6 wk. One-factor ANOVA was performed for statistical analysis. Differences between groups were analyzed by using Tukey's post hoc tests. Correlation water and food intakes was determined by using the parametric Pearson correlation analysis. Values are means ± SEMs, n = 6/group. Labeled means at a time without a common letter differ, $P < 0.05$. HF_x% 2'-FL, HF with x% 2'-FL (w/v) in drinking water; 2'-FL, 2-fucosyllactose.

Table S1. Diet composition of LF and HF diets[1]

Diet Ingredient	LF - D12450J		HF - D08091803B	
	g	kcal	g	kcal
Casein	200	800	200	800
L-Cystine	3	12	3	12
Corn Starch	401.29	2025	176.8	707
Maltodextrin 10	125	500	100	400
Sucrose	68.8	275	68.8	275
Cellulose (Solka floc, 200 FCC)	50	0	50	0
Soybean Oil	25	225	25	225
Lard	20	180	177.5	1598
Mineral Mix S10026	10	0	10	0
Dicalcium Phosphate	13	0	13	0
Calcium Carbonate	5.5	0	5.5	0
Potassium Citrate, 1 H20	16.5	0	16.5	0
Vitamin Mix V10001	10	40	10	40
Choline Bitartrate	2	0	2	0
FD&C Yellow Dye #5	0.04	0	0	0
FD&C Red Dye #40	0	0	0	0
FD&C Blue Dye #1	0.01	0	0.05	0
Total	1055.05	4057	858.13	4057
Protein, % kcal	20		20	
Fat, % kcal	10		45	
Carbohydrates, % kcal	70		35	
Energy, kcal/g	3.82		4.7	

[1] Diets prepared by Research Diets, Inc. HF, high-fat; LF, low-fat

Table S2. Primer sequences used for RT-PCR

Gene	Accession no.	Forward primers (5' to 3')	Reverse primers (5' to 3')
**GAPDH**	NM_008084.3	ACGGTCAGGTCATCACTATC	GATGCCACAGGATTCCATAC
**IL-1β**	NM_008361.4	ACAGATCGGCTCCTACTT	CGGGTCTGCTCATAGTAATG
**IL-6**	NM_001314054.1	AAACAGTCCAGGCTTCTC	ATGGCTGGGAACCATTAG
**MCP-1**	NM_011333.3	CGATGTCTAAGAGAGAAAGGG	GGAAACAGGTACCCACAAA
**PPARγ**	NM_011146.3	GAGCATCTCCCTCACAATTC	GGGTGCAGCGAACTTTAT

GAPDH, glyceraldehyde 3-phosphate dehydrogenase; IL-1β, interleukin-1 beta; Il-6, interleukin-6; MCP-1, monocyte chemoattractant protein-1; PPARγ, peroxisome proliferator-activated receptor gamma.

Table S3. ANOVA data summary

	F(DFn, DFd)	*P* value
**Two-way ANOVA** of body weight		
Interaction	F (30, 180) = 4.927	P<0.0001
Diet	F (5, 30) = 3.691	P=0.0101
Time	F (6, 180) = 258.4	P<0.0001
Two-way ANOVA of fat mass		
Interaction	F (5, 30) = 3.889	P=0.0078
Diet	F (5, 30) = 8.018	P<0.0001
Time	F (1, 30) = 84.02	P<0.0001
Two-way ANOVA of lean mass		
Interaction	F (5, 30) = 0.4699	P=0.7956
Diet	F (5, 30) = 0.1447	P=0.9801
Time	F (1, 30) = 19.94	P=0.0001
**One-way ANOVA (factor: diet)**		
Cumulative energy intake	F (5, 30) = 3.445	P=0.0141
Feed efficiency	F (5, 30) = 6.703	P=0.0003
c-Fos positive cells in the NTS	F (2, 13) = 2.131	P=0.1584
c-Fos positive cells in the AP	F (2, 13) = 1.291	P=0.3079
Cecal acetic acid level	F (2, 14) = 1.359	P=0.2887
Cecal butyric acid level	F (2, 14) = 4.593	P=0.0293
Cecal citric acid	F (2, 15) = 0.4953	P=0.6190
Cecal formic acid	F (2, 14) = 2.006	P=0.1714
Cecal glyceric acid	F (2, 15) = 6.461	P=0.0095
Cecal glycolic acid	F (2, 15) = 0.3647	P=0.7004
Cecal hexanoic acid	F (2, 14) = 0.4263	P=0.6611
Cecal isobutyric Acid	F (2, 15) = 1.917	P=0.1815
Cecal isovaleric Acid	F (2, 14) = 0.4263	P=0.6611
Cecal lactic acid	F (2, 14) = 7.456	P=0.0062
Cecal propionic acid	F (2, 14) = 0.1683	P=0.8468
Cecal pyruvic acid	F (2, 15) = 4.834	P=0.0240
Cecal succinic acid	F (2, 15) = 1.506	P=0.2535
Cecal valeric acid	F (2, 15) = 1.234	P=0.3192
Cecal indole-3-acetic acid	F (2, 15) = 1.234	P=0.3192
Cecal indole-3-butyric acid	The samples all had a standard error of zero.	
Cecal indole-3-lactic acid	Not detected	
Cecal indole-3-propionic acid	F (2, 14) = 0.1647	P=0.8498
Cecal serotonin level	F (2, 15) = 14.65	P=0.0003
Cecal IL-1β mRNA	F (2, 14) = 4.261	P=0.0359
Cecal IL-6 mRNA	F (2, 14) = 3.465	P=0.0599
MCP-1 mRNA in WAT	F (2, 15) = 5.299	P=0.0182
White adipocyte size	F (2, 11) = 10.33	P=0.0030
Plasma LBP level	F (2, 14) = 6.733	P=0.0089

Plasma Lcn-2 level	F (2, 13) = 2.751	P=0.1009
ORO-positive area in the liver	F (2, 14) = 0.3141	P=0.7355
Hepatic TG level	F (2, 15) = 1.463	P=0.2628
Hepatic PPARγ mRNA	F (2, 15) = 6.815	P=0.0078
Cumulative food intake (g)	F (5, 30) = 15.72	P<0.0001
Cumulative water intake (g)	F (5, 26) = 7.315	P=0.0002

Differences between groups were analyzed by using Tukey's post hoc tests. Differences were considered significant if $P < 0.05$. AP, area postrema; IL-1β, interleukin 1 beta; IL-6, interleukin 6, LBP, lipopolysaccharide (LPS)-binding protein; Lcn-2; lipocalin-2; MCP-1; monocyte chemoattractant protein-1; NTS, nucleus of the solitary tract; ORO, Oil Red O; PPARγ, peroxisome proliferator-activated receptor gamma; TG, triglyceride; WAT, white adipose tissue.
